# Metal Oxides Nanoparticles: General Structural Description, Chemical, Physical, and Biological Synthesis Methods, Role in Pesticides and Heavy Metal Removal through Wastewater Treatment

**DOI:** 10.3390/molecules28073086

**Published:** 2023-03-30

**Authors:** Zahrah Alhalili

**Affiliations:** Department of Chemistry, College of Science and Arts-Sajir, Shaqra University, Sahqra 17684, Saudi Arabia; zalholaili@su.edu.sa

**Keywords:** template synthesis approach, photocatalysis, adsorption, heavy metals, water treatment, pesticides, nanotechnology

## Abstract

Nanotechnology (NT) is now firmly established in both the private home and commercial markets. Due to its unique properties, NT has been fully applied within multiple sectors like pharmacy and medicine, as well as industries like chemical, electrical, food manufacturing, and military, besides other economic sectors. With the growing demand for environmental resources from an ever-growing world population, NT application is a very advanced new area in the environmental sector and offers several advantages. A novel template synthesis approach is being used for the promising metal oxide nanostructures preparation. Synthesis of template-assisted nanomaterials promotes a greener and more promising protocol compared to traditional synthesis methods such as sol-gel and hydrothermal synthesis, and endows products with desirable properties and applications. It provides a comprehensive general view of current developments in the areas of drinking water treatment, wastewater treatment, agriculture, and remediation. In the field of wastewater treatment, we focus on the adsorption of heavy metals and persistent substances and the improved photocatalytic decomposition of the most common wastewater pollutants. The drinking water treatment section covers enhanced pathogen disinfection and heavy metal removal, point-of-use treatment, and organic removal applications, including the latest advances in pesticide removal.

## 1. Introduction

### 1.1. Developmental History of NanoTech

**Figure 1:**
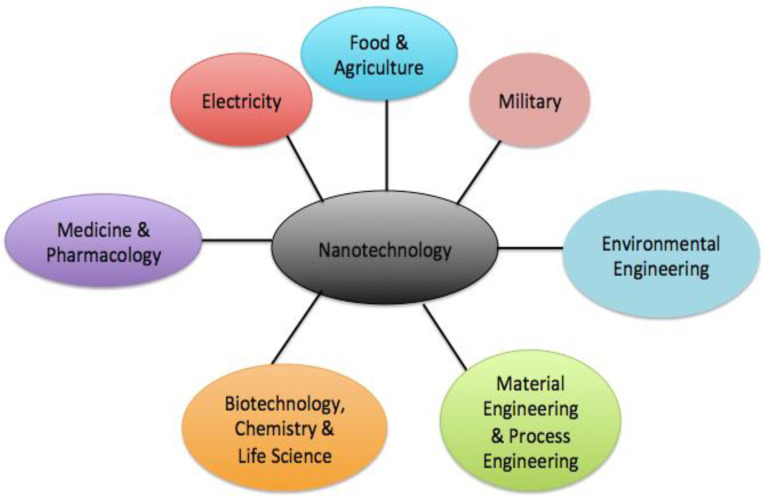
Application areas of nanotechnology. [1].

Nanotechnology (NT) refers to nanostructuring techniques like nanomanipulation, nanolithography, and nanomaterials (NMs). NMs generally refer to nanoparticles (NPs), nanotubes, nano films, etc. Nanotechnology connects diverse application sectors, including biology, biotechnology, chemistry, medicine, pharmaceuticals, food and agriculture, the environment, and electronics, among other industries (Figure 1). Nanomaterials are well described as nano-scale building blocks ranging from tiny atoms in groups to complex nanoparticles with sizes ranging between 1 and 100 nm. Over the years, some traditional materials have been replaced. The fundamental reason for this is that in general, NT provides NMs with different functionalities besides improved selectivity and reactivity compared to their bulk counterpart materials because of their high density and high surface area to volume ratio, in addition to high reactivity [1].

Increased reactivity mostly results in a variation of chemical properties when compared with the bulk material, such as improved chemical stability and dispersibility [2]. Saleh [3] highlighted that the main advantage of nanostructures is not only that they provide tunable chemical, physical, and mechanical properties, but also improved performance compared to their bulky counterparts. Their various properties were classified into optical, electrical, thermal, magnetic, and mechanical properties [4]. Nobel laureate and physicist Richard R. Feynman said during a lecture at the California Institute of Technology (USA) that “there’s plenty of space underneath” [5]. The concept of nanotechnology became fully operational in 1974 [6]. However, the term NT was introduced 25 years later via Eric Drexler [7].

### 1.2. Production Rates of NMs

Over the last 20 years, much information has been published on annual global production. Bao et al. [8] state that NP production increased by tons per year to 58,000 tons. Mueller and Nowack [9] noted in 2008 that the annual production of nano silver is about 500 tons. A significant increase in production is expected by the end of 2023. Nonetheless, this production rate does not include other NMs and primarily includes NPs. Generally, it is difficult to obtain comprehensive data on universal and individual production rates. Nagdi et al. [10] reported more detailed production rates for 10 different NM productions in Europe, America, Australia, and Switzerland. They showed that the production of nano-zinc oxide (ZnO), nano-titanium dioxide (TiO_2_), and nano-silica (SiO_2_) are the largest in the world, with an average annual peak production of 28,000 tons, 40,000 tons, and 55,000 tons, respectively. Sousa and Ribau Teixeira [11] noted that the rate of production of NMs in the EU in 2016 was around 1,615,000 tons. Giese et al. [12], also based on this data, estimate the annual growth rate to be 5%. Moreover, other scientists have estimated similar growth trends in Europe. Most of these products make Europe the leading producer of nanomaterials (more than 50%), then the United States (above 40%), and Asia (reached 10%) [11]. 

### 1.3. Classification of NMs

**Figure 2:**
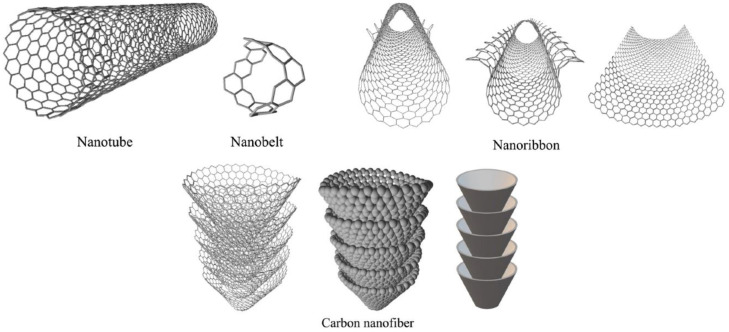
Different structures of nanotubes, nano ribbons, nano belts, and carbon nano fibers [1].

NMs can be classified by size, shape, and morphology. Their classification is highly dependent on their morphology and physicochemical properties [13]. When working with nanomaterials, various terminology is often introduced that can be misleading to inexperienced scientists and newcomers to this field of research. Therefore, a clear understanding is needed before addressing the more critical issues related to NT. Nanomaterials include nanoparticles, which are probably the most famous group as iron and silver nanoparticles hit the trade market, nano ribbons, nano films, nano fibers, nano liquids, nano spheres, nanotubes, nano rods, quantum dots, nanowires, and hollow spheres. The classifications of NMs are still based on their composition and dimensions. For example, the one-dimensional (1D) classification of nanomaterials includes thin nano films, nano sheets, and nano surfaces. Two-dimensional (2D) classifications include graphene sheets (wrapped in nanotubes) and nanowires, and three-dimensional (3D) classifications include graphite sheets, fullerenes, quantum dots, and dendrimers [14]. Examples of the most common NMs, like nano ribbons, nanotubes, and carbon nano fibers, are shown in Figure 2. With further classification, nanomaterials can be classified into single-phase solids (e.g., amorphous particles, crystals, and layers), multi-phase solids (e.g., matrix composites and coated particles), and multi-phase systems (e.g., colloids, aerogels, and magnetic fluid) [14]. Saleh [3] applied nanomaterials to zeolite- and silica-based NMs, metal-based NMs, carbon-based NMs, metal oxide-based NMs, polymer-based NMs, lipid-based NMs, ceramic-based NMs, etc. 

### 1.4. The Potential Applications of NMs

Nanotechnology has been given great attention due to its promising applications in different areas, including food production, cosmetic, treatment of diseases, biomedical, etc. [15]. In recent years, the focus on nanomaterials is clear due to their potential applications in different aspects of science and technology [16]. Nanomaterials can be classified into various types depending on their morphological structure, size, and other properties. These types are carbon-based nanomaterials, semiconductor-based nanomaterials, metal nanoparticles, polymer-based nanomaterials, and lipid-based nanomaterials [17]. Metal-based NMs include gold nanoparticles, silver nanoparticles, titanium oxide nanoparticles, iron oxide nanoparticles, zinc oxide nanoparticles, and other metal oxides [18]. They are used in different industrial fields, such as biomedical, pharmaceutical wastewater treatment, catalysis, and energy storage [17]. 

Iron oxide-based nanomaterials are extensively utilized in almost all areas because of their unique magnetic, electrical, chemical, and optical properties. They are utilized in various areas, including the biomedical field [19], catalysis [20], and wastewater treatment [21]. For example, Ebadi et al. [22] synthesized drug-based magnetic nanoparticles. In this technique, they utilized polymer (polyvinyl alcohol), zinc/aluminum-layered double hydroxide (Zn/Al-LDH), sorafenib drug as a coating and anticancer agent, and magnetic iron oxide nanoparticles as a core. The results from X-ray diffraction (XRD) and Fourier-transform infrared spectroscopy (FT-IR) analysis revealed the formation of the crystal structure core of the iron oxide. High-resolution transmission electron microscopy (HRTEM) indicated the size of the core iron oxide nanoparticles was about 30 nm. However, after surface modification of the iron oxide, the particle size increased to 95 nm. The results showed enhancement of anticancer activity against liver cancer and HepG2 cells when using drug-coated magnetic nanoparticles compared to the naked sorafenib drug. In addition, the prepared drug-coated magnetic nanoparticle scattering was nontoxic towards normal fibroblast 3T3 cells [22].

The optical properties of silver-based nanomaterials can be utilized in different sensor applications. Silver nanoparticles are remarkably effective at absorbing light, and their colour is related to their size and shape. Silver nanoparticles have been applied as antibiotic agents in textiles and wound treatment, medical equipment, and some appliances, such as refrigerators and washing machines [15]. For example, Rossi et al. [23] prepared silver nanoparticles by a chemical reduction method, and then the surface of the nanoparticles was modified with bifunctional mercaptoundecanoic acid (11MUA). The synthesized Ag NPs—11MUA was applied as a colourimetric sensor for the detection of Ni^2+^ metal ions in water [23]. 

In latest advances, metal oxide nanoparticles such as TiO_2_ NPs have been used for the photocatalytic degradation of different contaminants in wastewater. Exposure of pollutants to light and catalysts can oxidize them gradually to smaller molecular weights and some products such as water and carbon dioxide, and some anions such as NO^3−^ and Cl^−^ [18,24].

Platinum oxide-based nanomaterials have attracted great interest due to their prospective future applications in energy storage and as catalysts. Platinum (Pt) has excellent catalytic activity in various processes such as fuel cells, petroleum refining processes, and hydrogen generation [18]. For example, Nichols et al. [25] reported the preparation of platinum oxide nanoparticles deposited onto the surfaces of carbon nitride (C_3_N_4_) by thermal refluxing of C_3_N_4_ nanosheets and platinum chloride in aqueous media. The resulting products showed electrocatalytic activities towards hydrogen production reactions in acidic conditions. Additionally, it was noticed that when increasing the concentration of pt^4+^ species in the nanoparticles, the activity of the hydrogen evolution reaction improved [25]. 

## 2. Metal Oxide Nanoparticles

Metal oxide nanoparticles (MO_x_) are a fascinating class and diverse form of nanomaterials, mainly with applications in the chemical, physical, and materials sciences. Many metallic elements tend to react with oxygen under numerous conditions to form metal oxides with various structural forms [26]. Metal oxides with sizes in the nano scale range showed essential applications, such as fluorescence and optical sensors [27,28], catalysts [29,30], photovoltaic [31] and biomedicine [32], etc. Besides all these applications, nanomaterials have also been used like gas sensors [33] and anode materials for fuel cells [34]. The nanostructured metal oxide size and shape mainly affect the properties of surface-dependent nanomaterials such as optics, mechanics, electricity [35], magnetism [36], and catalytic ones [37]. Controlled adaptation of nanostructures to MO_x_ designing, besides achieving tunable properties, has attracted much attention to the exploration of an enhanced activity spectrum in biomedicine [38], industries [39], and other fields. This has given impetus to research efforts devoted to manipulating morphologically controlled nanomaterials with uniform size pores for desired properties. This can be determined by the fact that the magnetic behaviours of iron oxide nanoparticles exhibit size dependence, as the 55 nm Fe_2_O_3_ particles show ferromagnetic behaviour while the paramagnetic is obtained with particles with a size of 12 nm [40]. The synthesis of nanomaterials, with the fine size of the particle and homogeneous morphology, is an exciting area of scientific research in addition to concentrating on the synthetic methods of development to obtain the desired product. Countless techniques have been used to synthesize nanostructured metal oxides, such as co-precipitation, milling techniques, inert gas condensation, and lithography methods [41]. These conventional synthesis methods have the limitation of poor control over the morphology of the nanomaterials, thereby adversely affecting electronic conductivity and other properties [42]. 

The prepared materials’ ability with desired properties is mostly a very strenuous but remarkable task. One of the methods to realize the target product is a model-assisted synthesis methodology that includes the construction of microstructures to nanostructures with special chemical and physical properties varied from bulk materials [43]. There is much research in the literature and intentionally prominent articles on model-based nanomaterial preparation [44]. The evaluation reports presented modelled synthesis methods to fabricate various nanomaterials like 1D materials, porous films, transition metal oxides, etc. using different models. Liang et al. [45] noted template-assisted synthesis methods for 1D nanostructure synthesis using organic templates beside porous film materials as templates [45]. Wang Y. and colleagues [46] have demonstrated the fabrication of advanced structural materials using different compositions of planar and colloidal materials as templates through a layer-by-layer assembly method [46]. However, studies on the influence of hard and soft template-assisted synthesis methods on the morphological and surface properties of nanostructured MO_x_ have been established. Therefore, this report aims to provide a holistic and comprehensive review of modern model-assisted methods for the preparation of promising nanostructured MO_x_ from a wide variety of model materials. Template-assisted synthesis has become one of the most efficient methods to fabricate materials with controlled shape, size, and structural units [47]. The fixture is a central lattice structure that gives nano-scale sites for the synthesis of nano-scale materials. Template-mediated synthesis has resulted in the formation of well-defined morphological nanomaterials by controlling crystal growth at the nano scale. Removing the fixture from the product results in the creation of a cavity similar in shape and size to the removed fixture. This observation highlights the fact that the size and shape of the nanostructures depend on the size and shape of the template, which is confirmed by published research. Yourdkhani and colleagues [48] used anodic aluminum oxide as a substrate with a pore diameter of 200 nm and prepared a metal ferrite nanotube with a 200 nm diameter that supported substrate-matching by using liquid phase deposition combined with a template-assisted method [48]. Synthesis of TiO_2_ nanorods/nanowires for 1D nanostructures was carried out by Aisu and team [49] using porous anodic alumina (pore diameter 200 nm) [49]. Grote and his research team [50] also obtained a similar answer using an 80 nm diameter anodic aluminum oxide model and discovered the synthesis of MnO_2_ nanowires and nanotubes with a diameter of about 78 nm [50]. At present, template-assisted synthesis methods have attracted a great deal of interest besides emerging as excellent methods compared to template-free processes in nanomaterials preparation due to their iteration speed, high density, controlling the structure, and morphology, besides the size of the generated material. Based on the structure, models are divided into two types, named soft models and hard models [51,52]. From the findings in the literature, due to the hardness and unique structure, the use of rigid mould materials such as microporous zeolites, neutral materials, anodic aluminum oxide films, colloidal silicon spheres, etc. resulted in the correct shape and size. Products, in the case of flexible samples such as ammonium ions, amphoteric surfactants, liquids, and ionic product morphology control, are complicated because of their structural problems, as they are unstructured and have a fixed stiffness [53]. The mould material provides a large porous surface, besides acting as a structuring agent, leading to the formation of nanoparticles (micro to nanoscale), compared to the non-moulding process [54]. In the following, we briefly discuss different chemical methods for the fabrication of MO_x_ NPs.

**Figure 3:**
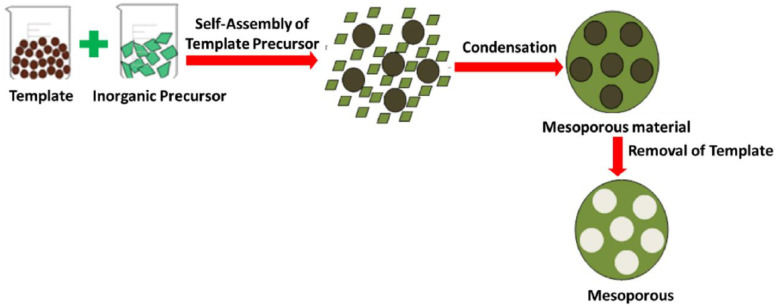
Synthetic procedure template-assisted for MO_x_ nanostructures. Reprinted with permission from Ref. [56]. Copyright 2022 Elsevier.

The synthetic process includes the synthesis of template-mediated MO_x_ (metal oxide) nanostructures through synthetic methodologies like sonication, hydrothermal, and electrochemical besides airless deposition, electricity, chemical vapour deposition, etc. The end step provides a pure, template-free nanostructured material that can be accomplished by chemical and physical methods such as sintering, melting, and etching [55,56]. Figure 3 exhibits the different steps of the model-based procedure for the forming of neutrals.

### 2.1. Preparation of MO_x_ Nanoparticles Using the Template-Assisted Method

Synthesis of model-assisted nanomaterials has emerged as an attractive method which offers the advantages of simple implementation, handling, and insensitivity to the conditions of the reaction. It has been observed that the presence of the substrate affects the grain size and crystalline MO_x_ nanomaterial’s phase/structure. In addition, template-assisted synthesis protocol allows for finer control of shape and morphology, besides size through limiting crystal growth in addition to nucleation during synthesis. Studies compiled in Table 1 demonstrate the ability of the mould material to influence not only the grain size variation but also the phase/crystal structure of the nanomaterials compared with that of the nanomaterials synthetic without mould. The robust synthesis approach supported by the model offers the potential to grow a multitude of morphologies such as nanowires, nanotubes, nanosheets, nanofibres, and more, depending on the material of the model, and usually consists of three stages. The first one may involve preparing a template like anodic aluminium oxide [56].

**Figure 4:**
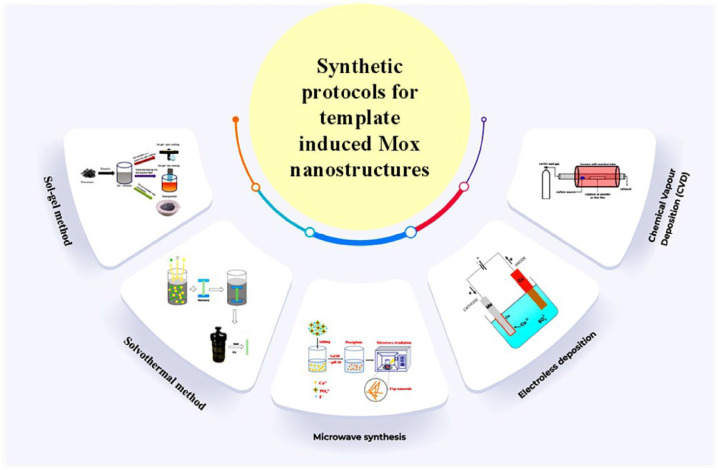
Synthetic protocols for the fabrication of template-assisted MO_x_ nanostructures. Reprinted with permission from Ref. [56]. Copyright 2022 Elsevier.

Model-assisted nanostructured metal oxide synthesis can be performed using synthesis procedures based on top-down and bottom-up approaches, as illustrated in Figure 4. Various methods like microwave, sol-gel, automated catalytic deposition, chemical vapour deposition, and hydrothermal/solvothermal have been performed to prepare nanostructured MO_x_ with targeted reactions. However, it can be inferred from the literature that in most situations, the template-assisted route is usually performed by applying sol-gel and hydrothermal synthetic methods [56]. 

### 2.2. Non-Biological Synthesis of MO_x_ NPs

#### 2.2.1. Sol-Gel Method

The sol-gel procedure is a popular synthesis method to fabricate MO_x_ nanostructures by modifying the precursor to a colloid (sol) and then gelling the reaction mixture. The gelation stage brings together the liquid and solid phases. Phase separation occurs by sedimentation or centrifugation and drying of the sample through sintering, concentration, or grain growth processes leading to the product formation. The final product is stable.

**Figure 5:**
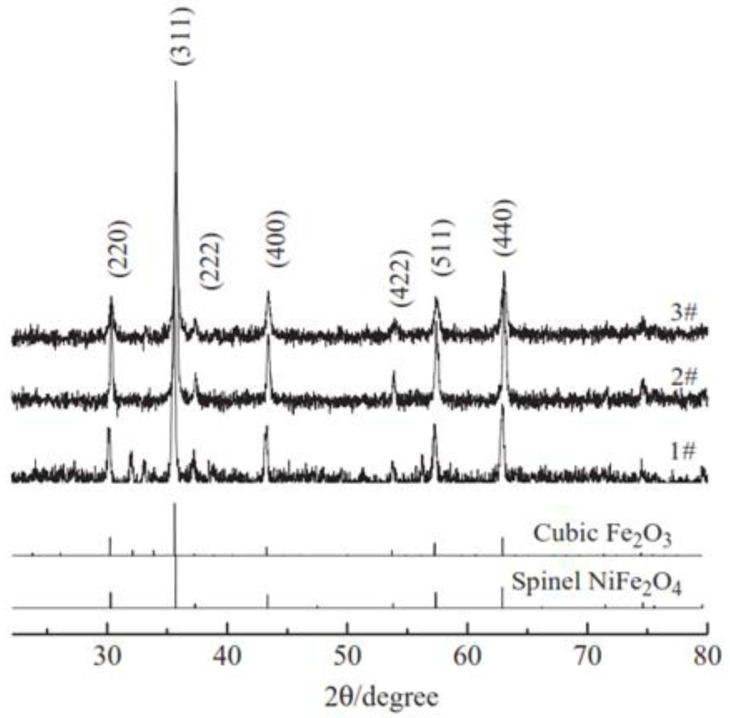
XRD patterns of the three samples synthesized, sample 1 (#1), sample 2 (#2) and sample 3 (#3). Reprinted with permission from Ref. [79]. Copyright 2014 Elsevier.

**Figure 6:**
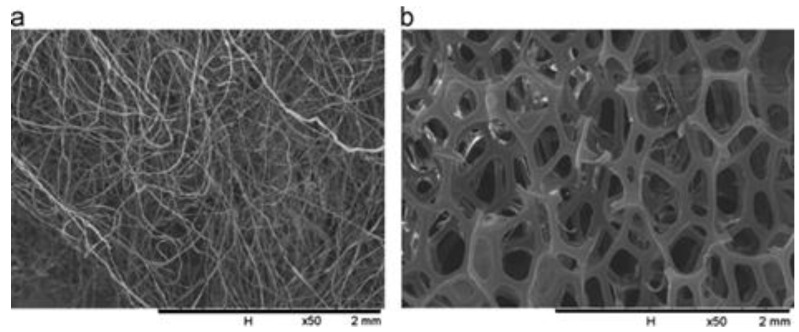
SEM images of the templates: (**a**) cotton and (**b**) sponge. Reprinted with permission from Ref. [79]. Copyright 2014 Elsevier.

The sol-gel technique is a widely utilized traditional and commercial method for producing numerous nanoparticles while affording excellent command regarding their size, high homogeneity and purity, and low temperatures. The secret is to create a homogenous sol from the precursors, transform it into a gel, remove the solvent from the gel, and then dry the gel. The equivalent metal alkoxide is often the molecular precursor; it is dissolved in either water or alcohol then heated and stirred to cause hydrolysis or alcoholises to form a gel [72]. Depending on the intended characteristics and use of the resultant nanoparticles, the right drying techniques are required. The wide size distribution of particles produced by sol-gel techniques is notable. A good example of MO NPs made by sol-gel includes the creation of ZnO nanoparticles using a modified sol-gel technique that produces particles that are 25 nm in size, which is smaller than what was previously reported for sol-gel methods, alternatively by standard sol-gel procedures [73]. Additionally, several additional MO NPs, such as Fe_2_O_3_ [74], MgO, and CdO [75], are usually created using the sol-gel method. In addition, the sol-gel synthesis method is one of the main protocols widely used by researchers for the nanostructured matrix of metal oxide-assisted syntheses like tungsten oxide nanoparticles, cerium oxide nanoparticles, TiO_2_, and NiO nanoparticles [76,77,78]. Li D-Y and team [79] synthesized nickel ferrite nanoparticles (NiFe_2_O_4_ NPs) by using the template-assisted sol-gel protocol. Three types of samples were produced: NiFe_2_O_4_ without template (sample #1), NiFe_2_O_4_-cotton template (sample #2), and NiFe_2_O_4_-sponge template (sample #3). It was found that the obtained NiFe_2_O_4_ NPs, tested by X-ray Diffraction (XRD), showed single phase spinel structures in a diameter of nanometers. The fabricated NiFe_2_O_4_ NPs with templates possess higher surface specific area and good magnetic properties, with no impurities in the structure presented in the XRD patterns (Figure 5). When utilizing sponge or cotton as templates, an enhanced saturation magnetization of 66.6 emu/g and coercivity of 137.7 Oe were obtained. Additionally, it was noticed that different crystalline sizes and grains were produced when applying different templates. The cotton and sponge templates were characterized by scanning electron microscopy (SEM). Figure 6 shows that absorbent cotton contains cellulose with a shape like twisted long fibres. On the other hand, Figure 6b illustrates the morphology of the sponge template, and it takes a honeycomb-like porous shape [79]. 

Nevertheless, the quality of the nanostructures obtained by the hard-based sol-gel procedure is relatively low compared to the soft-base sol-gel procedure in terms of morphologic characteristics [80,81]. 

#### 2.2.2. Solvothermal/Hydrothermal Procedure

The hydrothermal/melting procedure is applied to the crystalline materials synthesis, but it is mainly used to manufacture controlled and composed singular crystals under high temperatures beside pressure conditions. Due to the broad control over the morphology/particle size and reduction of particle aggregation, as well as the appropriateness for large-scale manufacturing and high purity, hydrothermal production of MO nanoparticles is widely used. However, there are lengthy reaction times and other post-processing procedures that are necessary. Hydrothermal synthesis is performed in an appropriate device called an autoclave [82]. In this device, a temperature grade is maintained between the ends of the growth chamber and the precursor (solvent) fed with water [83]. Typically, the synthesis includes adding a precipitating agent (such as NaOH) dropwise to an aqueous metal precursor solution before sealing the mixture within a Teflon-lined stainless-steel autoclave. This autoclave is then maintained at a consistent temperature, such as 80–200 °C, for a certain amount of time, such as 1–20 h, before going through several cleaning processes and annealing at the end. Synthesis takes place in a solvent more than water, a process known as solvent synthesis. Reaction conditions greatly affect the shape, size, and crystallinity of nanostructures [84]. The main advantages of this method are the simplicity and high crystallinity of the product, resulting in high yields [85]. Template-assisted hydrothermal synthesis has been used to produce metal oxides such as CuO/Cu_2_O composites, aluminum, titanium nanoparticles, Fe_2_O_3_, [86] NiO [87], and CO_3_O_4_ hollow spheres with precise size and shape distributions controlled to achieve crystallinity [88,89].

Abdelrahman et al. [54] reported successful template-assisted hydrothermal preparation of hydroxysodalite zeolite nanoparticles. It was found that different crystalline sizes ranging between 37.61 and 64.88 nm were obtained. The hydrothermal approach was applied with the use of aliphatic organic acids as templates or without using them. The aliphatic organic templates utilized in this study were oxalic acid dihydrate, tartaric acid, citric acid monohydrate, succinic acid, maleic acid, and ethylenediaminetetraacetic acid (EDTA). Different characterization techniques (HR-TEM, FE-SEM, FT-IR, and XRD) were used to characterize the prepared nanoparticles. The results from XRD show that the hydroxysodalite zeolite nanoparticles prepared without adding templates have the largest particle size of 64.88 nm, while the nanoparticles prepared by using EDTA have the smallest particle size of 37.61 nm. In addition, the results exhibited the efficiency of the prepared nanoparticles to eliminate zinc (II) ions from water with an adsorption capacity of 8.53 mg/g [54]. 

#### 2.2.3. Deposition by Electroless 

**Figure 7:**
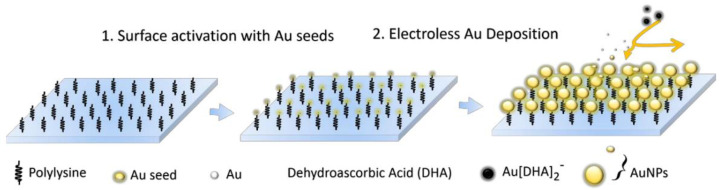
Schematic diagram of the synthesis of Au NPs films as uniform SERS-active substrates by the SMED approach [90].

**Figure 8:**
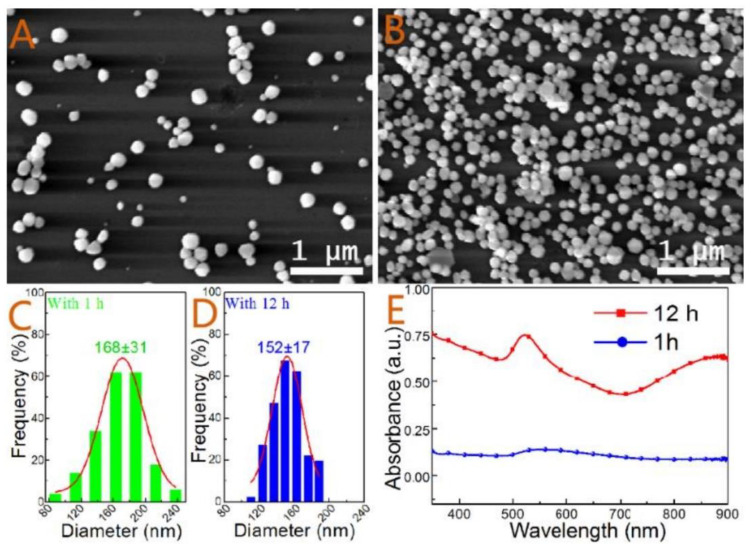
SEM images of the substrates with immobilized Au NPs without the activation process at various immersing periods: (**A**) 1 h and (**B**) 12 h. (**C**,**D**) Particle size distributions of Au NPs. (**E**) UV-Vis spectra of the substrates with immobilized Au NPs fabricated at different soaking times [90].

**Figure 9:**
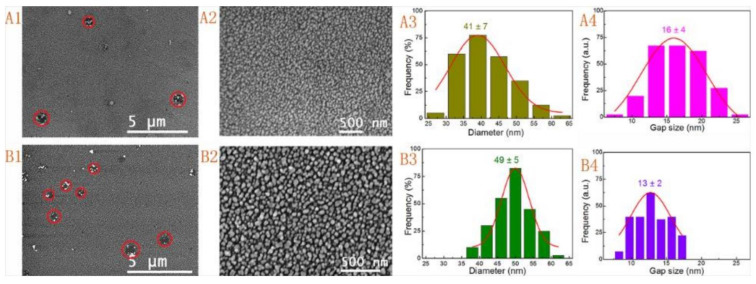
SEM images (**A1**,**A2**,**B1**,**B2**), size distributions (**A3**,**B3**), and gap size distributions (**A4**,**B4**) of Au NP films obtained by using seed I activation and with an immersion time of 30 min (**A1**–**A4**) and seed II activations with an immersion time of 12 h (**B1**–**B4**) [90].

Electroless deposition, also called chemical deposition or chemical reduction in situ, is a natural or autocatalytic reduction process created by Brenner and Riddell [90]. This mechanism involves depositing metal ions onto an activated surface in the presence or absence of an external field or mechanics such as an electrical current. Activation of the surface for deposition purposes was done by adding catalysts and reducing agents to solutions of metal ions. However, the solution must be continuously managed to ensure that its metal ion concentration remains constant. For example, Tang et al. [90] utilized a seed- mediated electroless deposition (SMED) technique (Figure 7) to synthesize gold nanoparticle films with uniform shapes and large surface areas to be used as substrates of surface-enhanced Raman scattering (SERS). Two types of gold seeds, named seed I and seed II, with different sizes, were fabricated. Typically, for preparation of seed I, 0.034 M of Na3 Citrate solution was added into 0.025 mM of HAuCl_4_ solution, followed by adding ice-cold 0.01 M of NaBH_4_ solution into the mixture quickly. For synthesizing seed II, 0.1 M of Na3 Citrate solution was added into 0.25 mM of HAuCl_4_ solution, followed by adding ice-cold 0.1 M of NaBH_4_ solution into the mixture. The two types of seeds were stirred in an ice bath until the reactions were accomplished. Then, the polylysine-coated glass slides were immersed in seed I or seed II to activate the surface of the polylysine-coated glass slides. The fabricated gold nanoparticles were characterized using different characterization techniques, including scanning electron microscopy (SEM) and ultraviolet-visible spectroscopy (UV-Vis). Figure 8A–E exhibits SEM images, particle size distribution, and UV-Vis spectra of the fabricated substrates without the activation process when applying various reaction times. It was noticed that only a few particles of Au settled on the substrate after 1 h of immersion time, and the size of gold nanoparticles was around 168 ± 31 nm. When increasing the soaking time to 12 h, the Au NPs density was increased. However, the size and standard deviation of the immobilized Au NPs were only 152 ± 17 nm. On the other hand, Figure 9A1–A4,B1–B4 shows SEM images and particle size distribution of the synthesized substrates with the activation process. The results indicate that the Au seed activation process is important to obtain Au NPs films with uniform shapes and large surface areas. When applying seed I activation, the average size of Au NPs and the mean interparticle gaps for the substrate with an immersing time of about 30 min were 41 ± 7 nm and 16 ± 4 nm, respectively (Figure 9A3,A4). Similarly, the values of the second substrate fabricated by adding seed II activations with an immersion time of 12 h were 49 ± 5 nm and 13 ± 2 nm, respectively (Figure 9B3,B4) [90]. 

In addition, the electroless deposition approach was used to fabricate other nanoparticles, such as Cu nanoparticles [91], Ag nanoparticles [92], Si nanoparticles [93], and structural nanoparticles such as MnO_2_, ZnO, and ZnO/CuO [94,95,96,97].

#### 2.2.4. Synthesis through Microwave 

**Figure 10:**
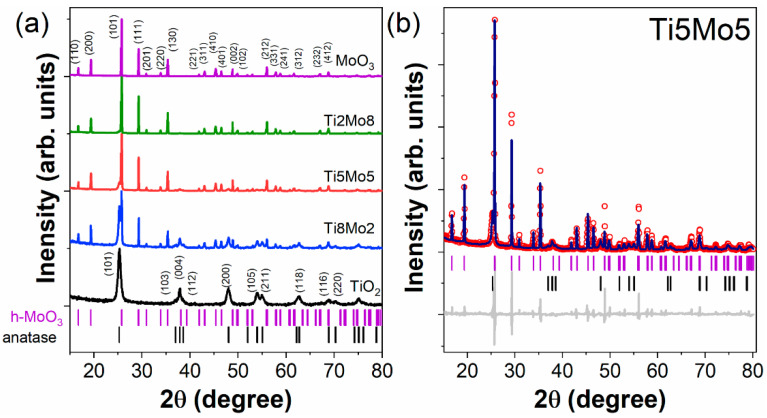
XRD patterns for TiO_2_-MoO_3_ composites. (**a**): Miller indices are shown on the pronounced peaks and (**b**): Rietveld refinement for selected Ti_5_Mo_5_ sample [99].

Synthetic fabrication of nanostructures of MO_x_ by microwave induction is a more environmentally friendly process performed under the microwave radiation effect (300 MHz and 300 GHz). The microwave synthesis of MO_x_ nanoparticles participates in the source metal decomposing, and the subsequent processing leads to the generation of metal oxides with sizes in the nanoscale range. This synthetic way provides advantages like reduced reaction times, significant control over the size and shape of nanomaterials, outstanding yield, and purity, as well as several shortcomings, as the method of microwave synthesis is not suitable for reaction enhancement and reaction monitoring cannot occur during synthesis. The synthesis method provides rapid degradation of metal salt precursors, and has also been explored for the matrix-based synthesis of a broad range of MO_x_ nanoparticles like graphene, oxide-zirconium, tin oxide nanoparticles, zinc oxide nanoparticles, CeO_2_, mixed oxides, and compounds of titanium and molybdenum with various sizes, compositions, and shapes [98,99,100,101]. For example, Kubiak and team [99] successfully reported the synthesis of the composite titanium oxide and molybdenum oxide (TiO_2_-MoO_3_ nanocomposites) by a template-assisted microwave approach. The prepared nanocomposites were characterized to observe their crystal phases by XRD and Raman spectroscopy, to examine their morphological structure via TEM, SEM, and HRTEM analysis, and also to monitor the variables of the porous structures through low-temperature N_2_ sorption. The prepared nanocomposites showed highly crystalline structures: anatase and hexagonal molybdenum trioxide (Figure 10). Overall, the template-assisted microwave method improved the incorporation of TiO_2_ NPs on the hexagonal MoO_3_ NPs surfaces. The prepared TiO_2_-MoO_3_ nanocomposites were utilized for electrochemical applications [99].

#### 2.2.5. Chemical Vapor Deposition (CVD)

Chemical Vapor Deposition (CVD) includes a precursor (solid or liquid) heating to form an active gaseous reactant, then transferring it into the reaction chamber. The CVD process uses non-toxic, volatile, and non-pyrophoric precursors according to the principles of green chemistry. Substrate exposure to volatile precursors causes reactions at the substrate surface and subsequent deposition to form the required product. The byproduct formed with the desired ones was damaged by the flow of gas through the reaction chamber. CVD techniques have been investigated for carbon nanotube synthesis [102] and MO_x_ nanostructures, such as ZnO nanocrystal powders, TiO_2_ nanowires, α-Fe_3_O_4_ nanoflowers, and iron oxide nanoparticles [103,104,105,106,107]. Du et al. [105] reported the synthesis of TiO_2_ nanowires on Ti_5_Si_3_ layers via atmospheric pressure chemical vapour deposition (APCVD). The prepared nanowires possess high density, crystalline phase, and hydrophilic properties. The obtained TiO_2_ nanowires were about 2–5 μm long with sizes ranging between 20 and 40 nm. The X-ray diffraction (XRD) showed the growth of TiO_2_ nanowires along the direction of [001] of tetragonal rutile TiO_2_ nanowires [105]. 

Interestingly, the morphological properties of nanostructured MO_x_ depend on the synthesis strategy, and these properties can be tuned by varying the synthesis method, reactant concentrations, and reaction conditions. Several limitations and challenges have arisen in introducing these nanomaterials into target applications, including a lack of understanding of the underlying modelling factors and procedures, the need for detailed analysis, etc., and increasing environmental friendliness through waste, by-products, and solvent consumption [56].

#### 2.2.6. Heterogeneous Catalysis

Catalysis is classified into homogeneous or heterogeneous catalytic processes [108]. Heterogeneous catalysts possess superior properties such as the ability to accelerate the reaction rates with inexpensive costs, the selectivity of the products and recycling of such catalysts leading to better sustainable production, and avoiding secondary contamination-related problems. In heterogeneous catalysis, the catalysts exist in different phases. For example, the reaction systems of heterogeneous catalysis contain liquid reactants with solid catalysts. Heterogeneous catalysts are involved in the manufacturing of greater than 80% of all chemical products worldwide. The demands for catalysts by industries such as energy generation, environmental remediation, chemical production, etc., have increased. Different types of materials can be used as catalysts, including metal and metal oxides, sulfides, nitrides, organometallic compounds, and enzymes [109,110]. A study reported by Valden et al. [111] showed gold nanoparticles supported on reducible oxides have an activity to oxidize CO to CO_2_ due to the effect of quantum size. Therefore, they can be used to minimize the CO levels in buildings by combining Au/TiO_2_ nano-powders with paint to cover the buildings’ walls [111]. In addition, it was found that vanadium oxide nanoparticles supported on metal oxides such as Al_2_O_3_, MgO, and ZrO_2_ exhibit activity to dehydrogenate alkanes to olefins because of the reduction cycle of vanadium oxide [112,113].

### 2.3. Bio/Green Synthesis of MO_x_ NPs 

Previous investigations have presented two methods for metal NP development: Top-down and bottom-up mechanisms. In a top-down process, nanoscale structures are etched onto the substrate with an electron beam, and then with a suitable etching and deposition process. The most commonly used top-down mechanisms are physical methods like evaporative condensation and laser ablation. This procedure uses the main resources. Most of the initial metal materials were vaporized by radiators, which are cooled at a sufficient rate using steep temperature gradients near the surface of the heating element. Rapid heating and cooling leads to highly concentrated and unstable NPs. Evaporative condensation is performed using an inert gas, while laser ablation through using a laser to target metallic materials in solution. Silver nanospheres (20–50 nm) may be prepared through laser firing using 800 nm femtosecond laser pulses in water. The main drawback is the poor surface structure. Such deforms can have a remarkable impact on the external interactions and physical properties of metallic NPs due to their high characterization relationships [114,115]. Chemical reduction was the most used approach via various carbon and mineral reduction intermediates. In general, numerous reducing intermediates such as sodium citrate, ascorbic acid, elemental hydrogen, sodium borohydride (NaBH_4_), polyols, toluene reagents, N, N-dimethylformamide (DMF), and poly(ethylene glycol) copolymer blocks body is used for reduction. Generation of metal ions in hydrous and non-hydrous solutions formed zero-valent metals and subsequently aggregated into oligomer clusters. These clusters eventually form metal colloid particles. Note also that most of these methods use protected intermediates (polymers) as stabilizers to avoid NP accumulation. The presence of polymers and surfactants (thiols, acids, amines, alcohols, etc.) affects the ability of interactions within the surface of the particle to stabilize particle growth and protect particles. Most of these methods are still in the development stage, as the purification and extraction of NPs ready for other applications still represent a significant obstacle [115,116].

Several radiation-assisted and mechanical approaches have been used to synthesize metallic NPs. Recently, the synthesis of green metal oxides through sonochemical methods has become popular. This is because it is the only way to promote the mixing of chemical components at the atomic level through reactions. An abnormal chemical reaction is applied due to foaming in an aqueous medium at 5000 °C temperature and 1800 kPa pressure. In 2021, Pérez-Beltrán [117] synthesized magnetic iron oxide nanoparticles using a high-energy sonication method, considering an amplitude of 2826 J and 1 min time as the key factors. This sonochemistry new one-minute green synthesis yielded 11 ± 2 nm nanoparticles, which were used in the bioreaction of mercury in water [102]. Another study conducted by Goudarzi [118] reported that *Dactylopius* bacteria could be used to ultrasonically synthesize copper oxide nanoparticles, followed by thermal decomposition at 60 °C to deliver drugs for breast cancer treatment [118,119].

In comparison to other wet chemical synthesis techniques, which use poisonous chemicals which can later be translated into the finished products, the green synthesis of MO nanoparticles has also drawn more attention because it uses environmentally friendly and non-toxic reagents, which has an impact on the usage of that kind of nanomaterials in pharmacological and other medical/biomedical applications. Other than the higher biocompatibility of the resulting nanoparticles, the benefits of green synthesis are based on the ability to regulate the morphology of the nanoparticles and cheaper costs, as well as the fact that the enzymes and proteins in the source materials work well as reducing and capping agents [120].

Microbial production of MO nanoparticles has been demonstrated to be a beneficial procedure because it is less hazardous than conventional high-pressure and chemical methods [121], as well as because the synthesis conditions have no negative effects on the bacteria. The widely used methods for the synthesis of metal NP were living organisms, including unicellular and multicellular ones. Some important descriptions include bacteria, fungi, plant extracts, diatoms, algae, yeasts, viruses, and some worms, such as earth-worms. Different sources of literature have developed dissimilar attempts to synthesize metallic NPs with biological artefacts. Bio-factories serve as non-toxic, clean, environmentally non-pollutant systems for synthesizing biocompatible NPs of a wide range of compositions, shapes, and sizes, in addition to physio-chemical properties. Most biological creatures use biopolymers as a template that helps stabilize nanostructures. Biofilm-forming agents enhance the NPs’ biocompatibility and prevent aggregation into clusters.

**Figure 11:**
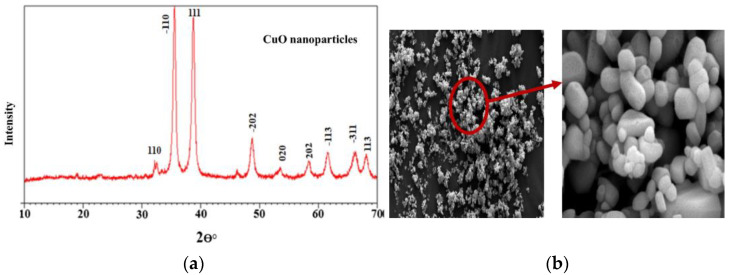
(**a**) XRD patterns of CuO NPs. (**b**) SEM images of obtained Cu NPs. Reprinted with permission from Ref. [123]. Copyright 2022 Elsevier.

However, plant extracts provide numerous enzymes and reduction agents that support direct NPs synthesis. Plant extracts are used in the photoreduction of metal ions as well as the creation and stabilisation of nanoparticles, making the green manufacturing of MO nanoparticles using them simple and quick, as well as ecologically sustainable. Table 2 shows list of some MO_x_ NPs synthesized from different plant samples with numerous applications [119]. The rate of the reaction is often slower during green synthesis, and the number of nanoparticles, shapes, and sizes that can be produced is constrained. This synthesis process is popular for the creation of MO nanoparticles for biological applications, focusing on different nanoparticles like zinc oxide nanoparticles [120] and Fe_2_O_3_ NPs [122]. Plant methods are very advantageous compared to microorganisms, as they do not require complex, separate, or multiple steps such as isolation, culture expansion, and preservation of culture. Moreover, plant-based synthesis is fast, inexpensive, and can be easily scaled up to produce large amounts of NPs. For example, Alhalili [123] reported the synthesis of copper oxide nanoparticles (CuO) from the *Eucalyptus globulus* plant. X-ray diffraction (XRD) results show the presence of diffraction peaks, indicating the successful formation of copper oxide with a monoclinic crystalline phase (Figure 11a). The shape of the prepared CuO NPs was like quasi-spherical particles (Figure 11b). These CuO NPs were utilized to remove methyl orange from aqueous media by adsorption technique [123]. The use of biological resources for the synthesis of metal NPs has increased exponentially in recent years [122].

## 3. Wastewater Treatment

Water pollution is classified as one of the biggest problems facing the world nowadays because the survival of species depends on suitable water for consumption. Water pollution causes negative consequences to the environment and human health, as well as to socio-economic progress. Numerous non-commercial and commercial procedures are applicable to combat this challenge, which is increasing due to advances in technology [130,131]. Nanotechnology has also proven to be one of the most advanced and best strategies for wastewater treatment. NP has high interaction, adsorption, and reactivity due to its small size and high surface-atom ratio [132,133]. They were suspended within hydrous solutions to act as colloids. These particles save energy due to their small size, which can ultimately lead to cost-effectiveness. NPs have a great advantage in treating water at great depths and in any place that has not been cleaned by conventionally available procedures [134,135,136]. Green nanomaterials are more likely to treat water contaminated with toxic metal ions, inorganic solutes, and organic and pathogenic microorganisms. Advanced research and commercialization of different nanomaterials (nanostructured catalytic membranes, nano-absorbents, biologically active NPs, nano-catalysts, biomimetic membranes, and molecularly imprinted polymers (MIP)) were performed to remove pathogenic bacteria, toxic metal ions, organic solutes, and inorganic substances from water [137,138]. The widespread usage of pesticides in the field of agriculture has had a significant impact on human health and the environment because these pollutants are not properly removed from the water. Pesticides also can have adverse effects on aquatic animals and humans because they are not completely removed from the aquatic environment by conventional wastewater treatment methods. Thus, processes such as heterogeneous photocatalysis and nano-composite adsorption have received considerable interest within the scientific society due to their unique properties, in addition to their ability to degrade and remove certain substances such as organic pollution, including pesticides. The most harmful pesticides are not those that are normally broken down/removed from the wastewater. Additionally, the potential of nano-catalysts and nano-absorbents to be aided in the removal of contamination of pesticide-containing wastewater is verified once they exhibit reduced band energy, which occurs over a distant range of wavelengths. Furthermore, due to the high affinity of the nano-catalysts supported with better charge separation, high removal and decomposition values were noted for these organic compounds. Therefore, the type of nanomaterials tested in this aim, magnetic or not, is characterized as conveyable nanomaterials with unique and potential properties useful in non-magnetic photocatalysts and homogenization and absorption of pesticides [139]. 

### 3.1. Decontamination Methods of Pesticides from Wastewater

#### 3.1.1. Nano Composites

Nanocomposite materials are multiphase materials prepared by dispersed and continuous phases and have at least one dimension at the nano scale [140]. The continuous phase (substrate) consists of a compound of polymer, ceramic, or metal. On the other hand, the dispersed phase (reinforced) is usually originated from fibrous materials [41,141]. In this view, nanoparticles of metal, chitosan, graphene oxide, and reduced graphene oxide can be applied as reinforcement that disperses upon the substrate to produce materials with elevated mechanical strength and properties, good optics, and large surface area [142,143,144]. Therefore, nanocomposites are easily used for wastewater and treatment of water, structural applications, and catalysis. Moreover, the nanocomposite synthesis effectively diminishes the surface energy and the aggregation tendency of magnetic nanoparticles enhancing their physicochemical stability [145]. Due to their non-expensive cost and ease of use, polymer and ceramic nanocomposites are extensively used in different applications. Polymer matrix nanomaterials may be prepared through in situ coating interlayer polymerization, prepolymer solution intercalation, or the sol-gel method [146], whereas ceramic matrix nanomaterials are synthesized through sol-gel synthesis, the powder method, the polymer precursor method, etc. [147].

Nanocomposite materials were synthesized to fuse individual properties and alleviate limitations, like thermal instability and physicochemical, thus expanding the range of applications [148]. Parallel reported the nanomaterials to exhibit behaviours distinct from those present at the micrometer grade, such as volume/area relationships and increased reactivity [149]. Another technique that is widely used to remove pesticides from wastewater is adsorption, in particular when using nanomaterials as adsorbents because of its simple operation, relative requirements, and low-cost energy demand [150]. In addition, nano-adsorbents are characterized by their high surface area, chemical/thermal stability, and their affinity for organic pollutants [151]. Although the effectiveness of nano-adsorbents in removing organic compounds is remarkable, there are limitations to the use of conventional materials, such as separation from aqueous media and recycling using nano-adsorbents and nano-catalysts [152]. Feng et al. [153] utilized a reheating preparation approach to fabricate Cu_2_V_2_O_7_/Cu_3_V_2_O_8_/g-C_3_N_4_ heterojunctions (CVCs). A higher surface area was obtained via the thermal etching process. The CVCs showed excellent adsorption and photocatalysis activities. It was found that CVC-2 comprising of 2 wt% of Cu_2_V_2_O_7_/Cu_3_V_2_O_8_ exhibited higher levels for the removal of different dyes and antibiotics; (96.2%) of methylene blue (MB), (97.3%) of rhodamine B (RhB), (83%) of ciprofloxacin (CIP), (86%) of tetracycline (TC), and (80.5%) of oxytetracycline (OTC) were removed via the adsorption and photocatalysis performance under visible light irradiation [153].

Lately, nanomaterials development, like nano-absorbent materials, has been the subject of various studies, due to their increased surface area and physicochemical stability. Furthermore, magnetic nanomaterials have been used as a good alternative to improve stability, structural properties, and reuse of the nano-absorbent [154]. The plants separate materials from the aqueous medium and greatly increase their reusability, resulting in a high adsorption capacity [155]. Furthermore, the same behaviour was observed for magnetic nanomaterials as nano-catalysts. The use of magnetic nano-catalysts allows material reuse, increases cost efficiency, and avoids further steps like centrifugation and filtration [156]. When combined with other magnetic nanoparticles (MNPs), photoactive compounds exhibit significant photocatalytic activity [157].

Furthermore, MNP incorporation on the surface of nanomaterials can improve the affinity and surface area for pollutants, which improves the adsorption capacity [158]. In addition, their magnetic properties do not have a fundamental role to make the adsorbent/catalyst be removed easily from the liquid phase after the reaction, and do not need centrifugation or other chemical compound usages that reduce processing costs [159,160]. Therefore, this work notes the nano-composites’ usage as nano-absorbents in addition to nano-catalysts in the degradation and removal of pesticides, in addition to the influence of different practical conditions on the efficiency of the processes of this program. Furthermore, this procedure also extends to magnetic nanomaterials, due to their potential flexibility within these processes. A research study conducted by Malektaj et al. [161] investigated the elimination of a pesticide called Malathion, which is an organophosphate with carcinogens effects, from water using iron oxide nanoparticles (Fe_3_O_4_ NPs) as a catalyst in the photocatalysis. The response surface approach was applied for the removal of Malathion. The Malathion removal is dependent on variables like pH, the concentration of Fe_3_O_4_ NPs, and the exposure time. The study concluded that the most effective conditions for the elimination of Malathion from aqueous media (82% was eliminated) were obtained with 0.4 g/L of Fe_3_O_4_ NPs, a contact time of approximately one hour, and a pH of 5 using ultraviolet radiation (Table 3) [162]. 

#### 3.1.2. Non-Homogenous Supported Nano Catalysts

**Figure 12:**
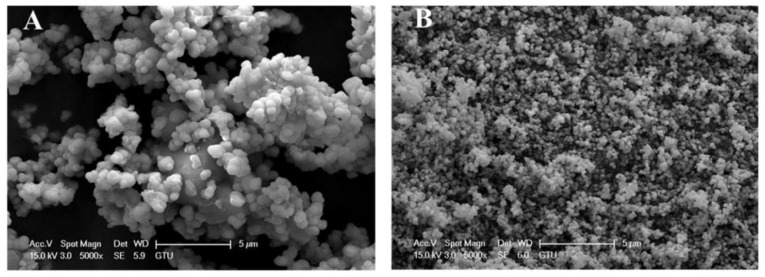
SEM images of TiO_2_ NPs for (**A**) at 1 h and (**B**) at 5 h [167].

Supported nano-catalysts composed of dispersed active nanomaterial over a material with less activity are known as catalyst supports. They are widely used due to their large surface area, considerable photocatalytic activity, and chemical/thermal stability in heterogeneous photocatalysis [162]. The application of nano-assisted catalysts (nano-composites) overcomes some of the disadvantages commonly encountered when isolated nano-catalysts are applied, including the agglomeration of nanoparticles because of their high surface energy and low dispersion in aqueous solution [163]. In addition, these catalyst aids elevate the binding between the catalyst surface and organic pollutants. Among the supported nano-catalysts used to degrade organic pollutants, Fe_2_O_3_, ZnO, CuO, and TiO_2_, supported on silica or zeolite, and are highlighted for their photo activity, remarkable catalysis, high surface area, and thermochemical stability [164]. In addition, the supported catalysts exhibit reduced band gap energies because of better charge separation that originates because of the presence of the support. Therefore, the ability to use this supported nano-catalyst even in the visible light spectrum complements its potential applicability in light-driven processes, especially catalysis, optically heterogeneous [165]. Furthermore, these nano-catalysts can easily be prepared through alternative synthetic procedures, such as leaf-based agents or plant extracts, microbial strains, phytochemicals, and industrial waste [166]. The following presents examples of green preparation of nanoparticles with no support material to be used for pollutant elimination from aqueous media. In the first example, Alhalili and Smiri [167] used aqueous extract attained from Aloe vera leaves to prepare titanium dioxide nanoparticles (TiO_2_ NPs) with photocatalytic properties. SEM images show the sphere-filled morphology of TiO_2_ NPs (Figure 12). The particle sizes were 83 ± 5 nm (2-dimensional), and 23 ± 2 nm (1-dimensional) after 1 h and 5 h of calcination, respectively. The prepared TiO_2_ NPs were utilized to remove Remazol Red Brilliant F3B (RR180) from the aqueous solution. It was found that the levels of RR180 dye decreased when time calcination increased. On one hand, TiO_2_ NPs (with size 23 ± 2 nm) showed a higher efficiency in photodegradation (100%) of RR180 dye under visible light irradiation for 1 h duration. On the other hand, TiO_2_ NPs with a diameter of 83 ± 5 nm presented a higher efficiency (100%) for up to 2 h duration [167].

**Figure 13:**
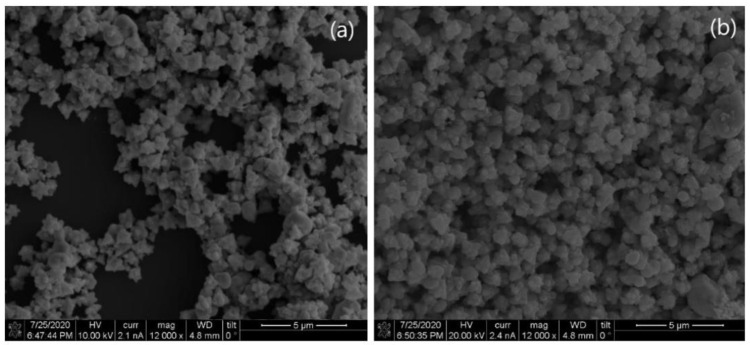
FE-SEM images of (**a**) CuO NPs and (**b**) NiO [168].

**Figure 14:**
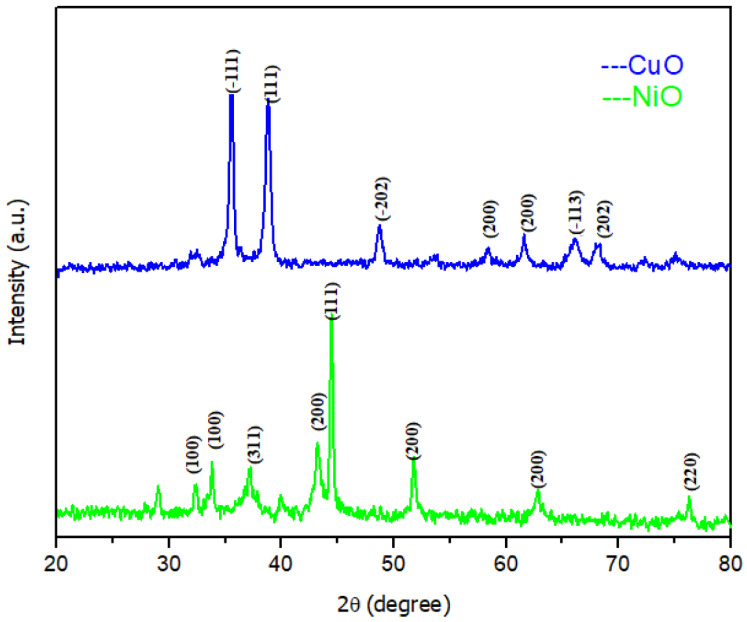
XRD pattern of CuO NPs and NiO NPs [168].

Another study reported by Iqbal et al. [168] showed a simple, ecofriendly, and stable synthesis of CuO and NiO nanoparticles using *Capparis decidua* leaf extract. The fabricated nanoparticles were analyzed using different techniques, including UV-vis, FE-SEM, XRD, and FT-IR. Figure 13 exhibits FE-SEM images for CuO and NiO NPs with flower and spherical-like morphologies of nanoparticles and homogeneous size distributions with an average size of approximately 900 nm for both CuO and NiO NPs. XRD patterns of both CuO and NiO NPs are presented in Figure 14. The XRD patterns have proven the face-centered cubic structure of CuO and NiO NPs, with average crystallite sizes of 11.23 and 16.75 nm, respectively.

In addition, the prepared nanoparticles were used to evaluate their efficiency to degrade the pesticide Lambda-cyhalothrin (L-CHT) in aqueous media. The results show that the photocatalytic removal of L-CHT pesticide was higher when using CuO photocatalyst (99% of L-CHL was removed) in comparison with NiO photocatalyst (89% of L-CHL was degraded). The reusability of CuO and NiO NPs against L-CHT was tested and can be for up to five runs. The results indicate the potential usage of CuO and NiO NPs photocatalysts for water treatment and the removal of pesticides [168].

Here is an example of the synthesis of nanoparticles embedded in other materials to be utilized for the removal of contaminants from water. Rahmanifar et al. [169] fabricated silver oxide nanoparticles embedded in chitosan (CS-AgO NPs) beads. The prepared composites were characterized using FT-IR, XRD, and SEM analytical techniques. SEM images for CS-AgO NPs beads showed a globular porous structure of CS-AgO NPs, indicating the production of CS-AgO NPs. The AgO NPs have a size of 64 nm. The AgO particles embedded in chitosan showed non-uniform spherical structures. Agglomeration of nanoparticles was also observed. This may be due to the existence of the capping agent.

The synthesized CS-AgO nanocomposites were evaluated for the degradation of the Permethrin pesticide. The reaction conditions include adsorbent dose, pH, and agitation time, and the primer concentrations of the pesticide were optimized. The results show that the chitosan-silver oxide nano-composites have higher adsorption efficiency compared to chitosan beads without AgO NPs. A total of 99% of the Permethrin pesticide was removed when using CS-AgO as adsorbent composites at room temperature with pH 7 and 0.5 g of the composites. The study concluded that CS-AgO NPs composites possess superior adsorption efficiency. The CS-AgO composite is nontoxic and biocompatible; therefore, can be used for the elimination of pesticides from aqueous solutions and water treatment applications [169]. 

#### 3.1.3. Adsorption

**Figure 15:**
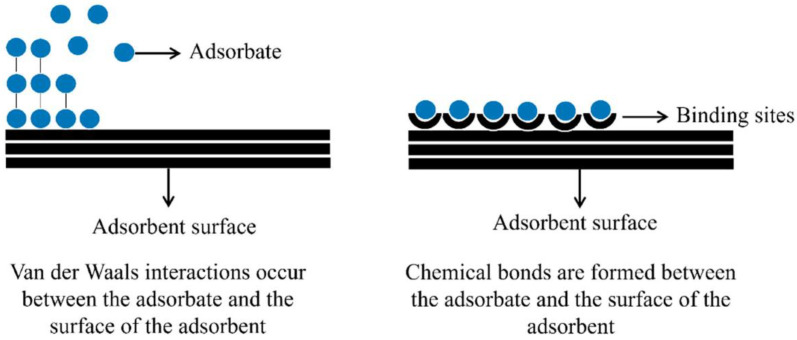
The adsorption process in single and multiple layers [170].

Adsorption is a surface phenomenon and physicochemical process in which a liquid (gas or liquid) reacts with a solid surface (adsorbent), causing a solute mass transfer from the liquid phase to a solid surface [155]. Within this system, the interaction degree of molecules and ions depends on pH, temperature, and concentration, as well as specific surface availability [170,171]. The attractive force between adsorbent and adsorbent can be classified into physical and chemical adsorption [172]. Chemisorption (chemical adsorption) participates in electron transfer and chemical bond formation. On the contrary, physical adsorption that originates from weak interactions between molecules includes electrostatic interactions, van der Waals forces, π–π bonds, and H bonds [173]. Without modifying the adsorption nature, certain parameters must be respected, such as the selectivity of the adsorbent, the uniformity and heterogeneity of the solid nanomaterials, and the adsorption rate that can be slow or fast. Furthermore, according to the process, the adsorbent can accumulate at the adsorbent surface in multilayer or monolayer form [174]. For further explanation, adsorption details are explained in Figure 15 in both single and multiple-layer models [170].

Adsorption is described as a comparatively non-complex technique besides its requirements as cheap and low energy; a wide range of compounds out of aqueous media can be effectively removed. However, the efficiency of the process depends on the intrinsic adsorbent properties, like pore volume, size of the particle, and chemical/thermal stability [174]. Dehaghi et al. [175] successfully synthesized chitosan-zinc oxide nanocomposite beads (CS–ZnONPs) composite via a polymer-based approach. 

**Figure 16:**
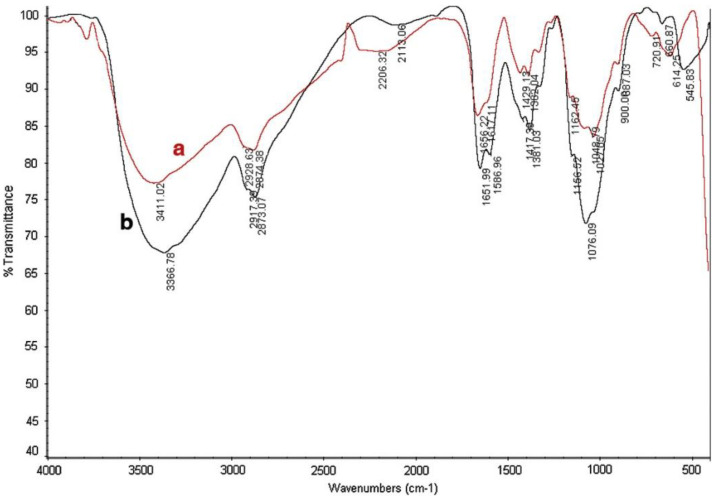
FT-IR spectra for (**a**) pure chitosan and (**b**) CS-ZnO NPs composite. Reprinted with permission from Ref. [175]. Copyright 2014 Elsevier.

**Figure 17:**
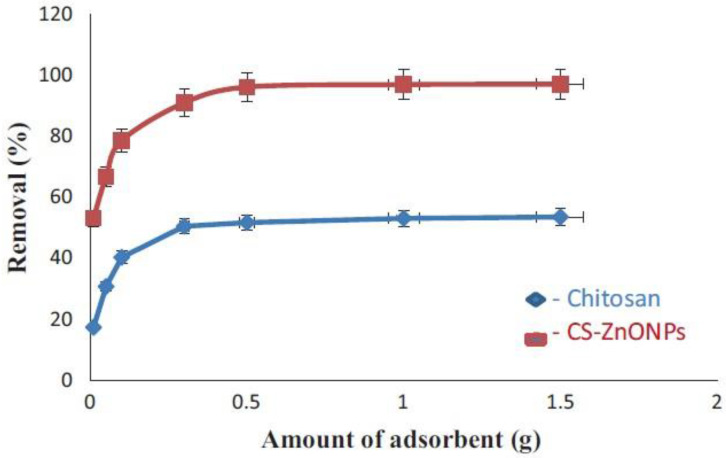
The effect of the amounts of adsorbent on removal percentage of Permethrin pesticide. Reprinted with permission from Ref. [175]. Copyright 2014 Elsevier.

The fabricated CS–ZnONPs composites were characterized using SEM, XRD, and FT-IR analytical techniques. In comparison with chitosan (FT-IR spectrum Figure 16a), FT-IR spectra for CS-ZnONPs composites (Figure 16b) show stronger peaks shifting at 3366 cm^−1^, indicating the attachment of ZnO NPs to amide groups of chitosan. The bands at 2917 and 2873 cm^−1^ are due to the asymmetry of CH_3_ and CH_2_ of chitosan while the adsorption band at 1651 and 1076 cm^−1^ are assigned to NH_2_ bending vibration. In addition, a new adsorption broad peak that appears at 580–400 cm^−1^ is attributed to O-Zn-O vibrations in Cs-ZnO NPs composite beads. The results indicate the formation of Cs-ZnO NPs composites. The ability of the prepared nanocomposite beads to remove Permethrin pesticide from aqueous media was investigated. The adsorption performance depends on different variables: adsorbent dose, pH, contact time, and the concentrations of the pesticide and CS-ZnONPs composite beads. It was found that the chitosan-zinc oxide nanocomposites have superior adsorption efficiency compared to chitosan beads without ZnO NPs (Figure 17). A total of 99% of the Permethrin pesticide was removed by 0.5 g of chitosan-ZnO nanocomposites at pH 7 [175]. 

## 4. Heavy Metals

Heavy metals, such as mercury, arsenic, cadmium, lead, and copper, are chemicals that are five times denser than water and have negative impacts on both plants and animals [176]. These are substances with qualities like heat conductivity, current flow, and a lustrous surface. They are present everywhere in nature, but where they are concentrated might change. Many organs in both plants and humans, in very small amounts, need these heavy metals to perform their normal functions. However, when their concentration exceeds the safe limit, they become hazardous. They are on the list of significant contaminants and worries about them are growing daily [177,178]. Pesticides, insecticides [179], municipal sewage, industrial effluents, mining, weathering, and erosion caused by wind and water are a few significant sources that make it easier for them to enter the environment [180].

While some of these metals, like iron, are vital to plants and animals biologically, others, like lead, chromium, etc., are not as crucial to living things and may even be poisonous when present for prolonged periods. Through stomata or the water absorbed by root hairs, they get access to the plant body. They are introduced into the human body through skin contact, air inhalation, and water and food [180].

Water is one of the simplest and most significant routes for heavy metals to enter the bodies of plants and animals. As a result, worries regarding heavy metal contamination of water are growing. Considering this, the majority of research focuses on water purification. Due to the toxicity of heavy metals and other negative impacts they have on living things, increased heavy metal contamination reduces the beneficial uses of water. Due to their inherent toxicity and consequent toxicity, heavy metals have received a lot of attention recently [181,182]. These heavy metals may have harmful, stimulatory, or inhibitory effects on biological reactions. Different problems are brought on by these heavy metals in both plants and animals. They cause different diseases in people, including cancer, Parkinson’s, Alzheimer’s, bone mineralization, effects on DNA and RNA, and problems with the reproductive system. Children with mental disorders are at higher risk of heavy metal toxicity than adults with dementia, major organ dysfunction, depression, eyesight problems, and emotional disturbances [183,184]. To limit the danger posed by these heavy metals in water, numerous strategies have been devised. These approaches can be classified into two categories: traditional methods based on nanotechnology, such as ion exchange, reverse osmosis, electrolysis membrane process, coagulation, precipitation, adsorption, and chemical reduction [185]. Demiral et al. [186] have reported the synthesis of activated carbon obtained from grape bagasse by the chemical activation approach. The prepared product was utilized to remove Cu (II) from aqueous solutions. Different characterization techniques such as scanning electron microscopy (SEM) and Fourier transform infrared spectroscopy (FTIR) have been used to characterize the adsorbent [186]. 

**Figure 18:**
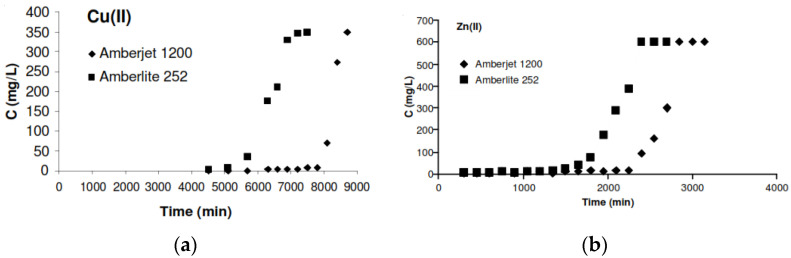
Comparison between the performance of Amberjet 1200 and Amberlite 252 for removal of (**a**) copper (II) and (**b**) zinc (II) [187].

Another study conducted by Budak T.B. [187] applied an ion exchange method to remove heavy metals from an aqueous medium. In this study, two strong acid cation-exchanger resins, Amberlite 252 and Amberjet 1200, were utilized to remove copper (II) with a concentration of 350 mg/L and zinc (II) with a concentration of 600 mg/L, with a flow rate of 2.5 mL/min and 35 g of adsorbent. The results demonstrate that the Amberjet 1200 performed better than Amberlite 252 and adsorbed these two metals (copper (II) and zinc (II)) from synthetic rinse water (Figure 18) [187].

## 5. The Potential Applications of Metal Oxides Nanoparticles in Water Treatment

Water is an essential need for life on our planet. However, obtaining safe and clean freshwater is rare due to population growth over the world and climate change effects. Therefore, there is a need to reuse treated wastewater. Strict regulations for reusing and discharging effluents are applied in wastewater treatment industries [188]. Wastewater is a new prospective source for obtaining clean water that can be utilized for all purposes. It is expected that the reused wastewater would provide better quality and would be cleaner than the existing water [189]. 

Water is polluted by different contaminants, such as heavy metals, pesticides, herbicides, pharmaceuticals, pathogens, etc., which influence living creatures. Poor water quality causes direct and indirect diseases [190,191]. Different conventional water treatment technologies including adsorption, reverse osmosis, electrochemical, ion exchange, oxidation, sedimentation, chlorination, and membrane filtration are utilized for water treatment. However, these traditional techniques have limitations. For example, reverse osmosis is an efficient process for wastewater treatment, but its efficiency to remove bacterial and chemical pollutants is limited, and it is a costly technique. In addition, the elimination of some metals and minerals using the separation by sedimentation technique is not efficacious. Also, the chlorination step utilized for pathogens and bacteria removal leads to unwanted taste and smell in treated water. 

Therefore, novel water treatment technologies are essentially required to overcome the limitations of those conventional methods, in particular the removal of micro or nanoscale contaminates. Nanotechnology has emerged as a potential solution for water and wastewater treatment. It exhibits the utilization of nanomaterials, including nano-adsorbents, nano-metal oxides, and photocatalysts in the processes for treating water. 

Nanomaterials have enormous physical, chemical, and biological properties, including a high surface area to the ratio, small size, cheap, stable, easy synthesis approaches, and ecofriendliness. These unique properties make nanomaterials able to remove different residual pollutants. They can effectively adsorb organic and inorganic contaminants from wastewater, which is not easy when using other conventional technologies. Additionally, nanoparticles do not cause the production of byproducts during manufacturing processes. Various types of nanomaterials, such as carbon nanotubes, silver nanoparticles, iron nanoparticles, and copper nanoparticles, have been used for removing different contaminants from water [190,191]. 

Metal oxide nanoparticles have been extensively utilized to remove different organic and inorganic contaminants from wastewater [192]. Table 4 presents some metal oxide nanoparticles used for the removal of different contaminants from wastewater. 

A study conducted by Jiang et al. [193] investigated the performance of adsorption and membrane filtration-based nanotechnology for wastewater treatment. It was found that the adsorption method is simple and could remove organic and inorganic contaminants efficiently in comparison with membrane filtration. The adsorption performance was assessed by the pore structure of the adsorbent and the interactions between the adsorbent and the pollutants [193]. 

Functionalizing the surface of metal oxide nanoparticles and exploiting their unique properties, besides controlling experimental conditions, enable them to interact with contaminants and then remove them from wastewater. 

Another research study was performed by Le et al. [194]. In their study, magnetic graphene oxide-chitosan composite beads (MGOCS) were prepared to investigate the removal efficiency of reactive blue 19 (RB19) and nickel Ni (II) ions. The effects of different experimental parameters including pH, the dosage of adsorbent, and contact time were examined. In the adsorption method, pH value is important and could affect the surface charge properties and the interactions between adsorbent and contaminants. The results showed adsorption capacities of MGOCS were 102.06 mg/g for RB19 and 80.48 mg/g for Ni (II) [194].

The common technologies applied to remove metal ion pollutants from wastewater include adsorption, precipitation, and photocatalysis. Qing et al. [195] investigated the adsorption actions of gold (Au), silver (Ag), and palladium (Pd) on titanium dioxide nanoparticles (TiO_2_ NPs) at low concentrations using inductively coupled plasma atomic emission spectrometry (ICP-AES). The results showed that the adsorbent TiO_2_ NPs had adsorption capacities of 22.63, 14.06, and 11.82 mg/g for Au, Ag, and Pd, respectively [195]. 

Mesoporous silica nanoparticles have been used in adsorption and catalysis processes due to their properties such as large surface area, simple surface functionalization, and large pore volume [196]. Sachan et al. [197] reported the preparation of SiO_2_ NPs by using *Saccharum ravannae* (SRL)*, Saccharum officinarum* (SOL), and *Oryza sativa* (OSL) leaves. The synthesized nanoparticles were utilized as adsorbents to remove Pb (II) and Cu (II) heavy metal ions from synthetic wastewater. The adsorption capacities for Pb (II) and Cu (II) were 140.06 mg/g and 149.25 mg/g for SRL SNPs, 338.55 mg/g and 179.45 mg/g for SOL SNPs, and 334.7 mg/g and 274.02 mg/g for OSL SNPs. Garg et al. [198] reported the biosynthesis of silica-based zinc oxide nanocomposites to be used as an adsorbent to remove Ni^2+^, Cd^2+^, and Cu^2+^ heavy metal ions from synthetic water. The adsorption capacity of silica-based zinc oxide nanocomposites was 32.53, 32.10, and 30.98 mg/g for Ni^2+^, Cd^2+^, and Cu^2+^, respectively [198]. 

On the other hand, Arshad et al. [199] prepared double-functionalized graphene oxide (GO) embedded in alginate biopolymer matrix (*ff*GOCA) beads as the adsorbent to remove heavy metal ions, polyaromatic hydrocarbons, and phenols from real refinery wastewater. The adsorption capacities of the *ff*GOCA beads were 116.7 mg/g for naphthalene, 105.2 mg/g for p-cresol, 111.1 mg/g for phenol, 102.04 mg/g for fluorene, 588.2 mg/g for Pb, 476.19 mg/g for Cd, and 434.7 mg/g for Hg. The prepared *ff*GOCA beads indicate the potential usage of these beads to remove different organic and inorganic contaminants from real wastewater [199]. 

Saien et al. [200] investigated the removal of aliphatic and aromatic organic compounds (2-methoxy-2-methylpropane, cyclopropane, benzaldehyde, methyl-tetrabutyl ether, phenol, 2,3,5,6-tetramethylphenol, naphthalene, xylene, 2,4-dimethylphenol (xylenol), 2,5-dimethyl-3-ethylphenol, octamethylcyclotetrasiloxane, tetradecane, 4-chloro-3-methylphenol, and 3-tert-butylphenol) from real petroleum refinery wastewater by using titanium dioxide nanoparticles as the photocatalyst in the ultraviolet UV irradiation process. The maximum degradation performance of more than 78% of the organic contaminants was obtained after 120 min of using the UV/TiO_2_ NPs system. The optimal experimental conditions applied were 100 mg L^−1^ of the catalyst concentration, pH of 3, and temperature of 45 °C. The results indicated an efficient degradation of different organic contaminants in real petroleum refinery wastewater [200]. 

Bernabeu et al. [201] utilized a real wastewater sample. It was collected from the outlet of a plant in the southeast of Spain. The collected wastewater was analyzed before treatment. The sample had large amounts of nine contaminants (trimethoprim, ofloxacin, enrofloxacin, clarithromycin, acetaminophen, diclofenac, caffeine, thiabendazole, and carbamazepine). After that, the wastewater samples were treated using TiO_2_ NPs/solar photocatalysis system. Large amounts of the pollutants were removed, and only small traces remained after 3 h of treatment. Additionally, it was noticed that about 100% of faecal bacteria were removed from wastewater after 1 h of treatment [201].

Micro-pollutants (MPOs) present in wastewater can be adsorbed by nanoparticles that are used to remove such contaminates. The properties of both MPOs and NPs can be changed after the interaction between them. This may lead to a change in their transport, fate, toxicity, size, surface, stability in water, conductivity, bioavailability, and other properties. The environmentally ambient conditions such as temperature, pH, and ionic strength with the properties of both NPs and MPOs control the interaction mechanism between the NPs and the contaminants [202]. 

**Table 4:** Various nanoparticles are utilized for the removal of different contaminants from wastewater.

Nanoparticles	Synthesis Method	Shape	Diameter (nm)	Contaminants	Removal%	Ref.
Iron oxide NPs	Tangerine peel extract	Spherical	50 nm–1 μm	Cd (II)	90%	[203]
Zinc oxide NPs	Co-precipitation	Spherical	374.1–730.2	Phenol	100%	[204]
Fe_3_O_4_ @1	combining Fe_3_O_4_ and polyoxometalate.	Mostly spherical	19.1	methylene blue (MB), rhodamine B (ChB), safranine T (T), gentian violet (GV), fuchsin basic (FB)	96.9%, 96.3%, 89.1%, 96.1%, and 94.5%, respectively.	[205]
Titanium oxide NPs	Peepal leaf extract	Agglomerated particles	11–91	Methylene blue, methylene orange	64% and 28%	[206]
Copper oxide NPs-1	Mint leaf extract	Mostly spherical	~150	Cd (II), Ni (II) and Pb (II).	18%, 52.5%, and 84%	[207]

## 6. Conclusions

Nanotechnology has emerged with an attractive potential future because it provides better solutions for the problems that exist in different sectors, such as biomedical, pharmaceutical, medicine, environmental, agricultural, and industrial areas. 

Nanomaterials have been widely utilized for the elimination of different organic (dyes, pesticides, etc.) and inorganic (ions, heavy metals, etc.) contaminants from wastewater due to their unique chemical, physical, and biological properties compared to bulk materials.

This review focuses on the synthesis of metal oxide nanoparticles using different preparation approaches such as the template-assisted method, sol-gel method, chemical vapour deposition method, hydrothermal/solvothermal approach, and deposition by the electroless method. Template-assisted synthesis offers a greener and more promising protocol compared to traditional synthesis methods such as sol-gel and hydrothermal synthesis, and endows products with desirable properties and applications. Template-assisted synthesis enables the synthesis of metal oxide nanoparticles with controlled structures and morphologies with desired properties. It supplies a comprehensive overview of nowaday developments in the areas of drinking water treatment, wastewater treatment, remediation, and agriculture. 

Green synthesis of metal oxides using biological resources (plant extracts, microbial) was also discussed. The biosynthesis of NPs has attracted great interest due to its non-toxic nature. Plant-based synthesis is preferable in comparison with microorganisms because their synthesis steps are simple and there is no need for further steps such as culture expansion and preservation of culture as in microorganisms. Moreover, plant-based preparation is fast, inexpensive, and can be easily scaled up to produce large amounts of NPs.

The drinking water treatment section covers enhanced pathogen disinfection and decontamination methods of pesticides from wastewater, including the use of nanocomposites, adsorption process, photocatalysis, and heavy metal removal. For example, 82% of the Malathion pesticide was eliminated using iron oxide nanoparticles (Fe_3_O_4_ NPs) as a catalyst in photocatalysis. The pesticide removal from wastewater is dependent on factors like pH, the concentration of the adsorbent and adsorbate, and the contact time. 

In addition, wastewater is a promising source for obtaining safe and clean water that can be utilized for all uses. The reused wastewater would provide better water quality and would be cleaner than the present water. 

The promising potential applications of metal oxide nanoparticles for water and wastewater treatment were highlighted. The most popular methods used to remove pollutants from wastewater include adsorption and photocatalysis processes. They are efficient processes for the removal of toxic contaminants. 

Various metal oxide nanoparticles, such as titanium dioxide nanoparticles, mesoporous silica nanoparticles, zinc oxide nanoparticles, iron oxide nanoparticles, and copper oxide nanoparticles were used in literature to remove organic and inorganic pollutants from wastewater. Metal oxide nanoparticles possess exceptional properties such as high surface area, small size, and stability, enabling them to remove different contaminates from wastewater by the interaction between the particles and the pollutants. 

## Figures and Tables

**Table 1:** Summary of matrix-assisted synthesis impact on grain size beside crystal structure of selected MO_x_ nanomaterials. Reprinted with permission from Ref. [56]. Copyright 2022 Elsevier.

Formed MO_x_ NPs	Particles Size without Template	Particles Size with Template	Phase/Crystal Structure		Ref
			With Template	Template-Free	
ZnO	20 nm	28 nm	Wurtzite	Hexagonal	[57,58]
CeO_2_	4 nm (105)	15–36 nm	cubic fluorite	face centred cubic	[59,60]
Co_3_O_4_	10 nm	20–37 nm	Cubic	face centred cubic	[61,62]
In_2_O_3_	15 nm	20–30 nm	Cubic	rhombohedral	[63,64]
SnO_2_	6–15 nm	18 nm	tetragonal rutile	tetragonal rutile	[65,66]
TiO_2_	30–40 nm	100 nm	anatase	Rutile	[67,68]
Mn_3_O_4_	5 nm	25 nm	tetragonal	hausmannite tetragonal	[52,69]
NiO	8 and 26 nm	31 nm	hexagonal	face-centred cubic	[70,71]

**Table 2:** List of some MO_x_ NPs synthesized from different plant samples with numerous applications [119].

Plant	Source of NPS	Metal Oxide	Size	Application	Ref.
*Ficus carica*	Leaf	Fe_3_O_4_	43–57 nm	Antioxidant	[124]
*Azadirachta indica*	Leaf	CuO	NA	Anticancer	[125]
*Peltophorum pterocarpum*	Leaf	Fe_3_O_4_	85 nm	Rhodamine degradation	[126]
*Terminalia chebula*	Seed	Fe_3_O_4_	NA	Methylene blue degradation	[81]
*Punica granatum*	Peel	ZnO	118.6 nm	Antibacterial property	[127]
*Lactuca serriols*	Seed	NiO	NA	Degradation of dye	[128]
*Vitis rotundifolia*	Fruit	CoO	NA	Degradation of acid blue dye	[129]

**Table 3:** Experimental results were designed using the central composite design (CCD) approach [161].

Malathion Removal%	Contact Time (min)	pH	Malathion Concentration (mg/L)	Nanoparticle Value (g/L)	Experiment Run
21.7	50	9	35	1.05	1
23	100	9	35	0.55	2
64.5	100	5	85	1.05	3
71.33	125	3	35	1.05	4
74	100	7	60	0.3	5
17	100	9	85	0.55	6
82	60	5	35	0.4	7
57.7	25	5	110	1.3	8
3.4	75	11	60	0.80	9
72	50	7	100	0.80	10

## Data Availability

Relevant data applicable to this research are within the paper.
